# Nothing About Us Without Us: Involving Youth Living With HIV in a Virtual Advisory Board

**DOI:** 10.1016/j.jadohealth.2023.06.028

**Published:** 2023-09-04

**Authors:** Autumn B. Chidester, Catherine J. Johnson, Hueylie Lin, Ruby Viera Corral, Susan Kools, Karen S. Ingersoll, Rebecca A. Dillingham, Ank E. Nijhawan, Anna G. Taranova, Barbara S. Taylor

**Affiliations:** a Division of Infectious Diseases, Department of Medicine, University of Texas Health Science Center at San Antonio, San Antonio, Texas; b Research and Information Management Department, University Hospital Systems in San Antonio, San Antonio, Texas; c School of Nursing, The University of Virginia, Charlottesville, Virginia; d Professor of Psychiatry and Neurobehavioral Sciences, Department of Psychiatry and Neurobehavioral Sciences, Center for Behavioral Health and Technology, University of Virginia Health, Charlottesville, Virginia; e Department of Medicine, The University of Virginia, Charlottesville, Virginia; f Division of Infectious Diseases, Department of Internal Medicine, University of Texas Southwestern Medical Center, Dallas, Texas; g Department of Research and Healthcare Innovation, University Hospital Systems in San Antonio, San Antonio, Texas; h Professor of Infectious Diseases, Assistant Dean for the MD/MPH Program, Division of Infectious Diseases, Department of Medicine, University of Texas Health Science Center at San Antonio, San Antonio, Texas

**Keywords:** Youth advisory board, Community advisory board, Youth living with HIV, mHealth, Intervention development, HIV, Community engagement

## Abstract

**Purpose:**

We adapted a traditional community advisory board to the needs of youth living with HIV (YLWH), resulting in a virtual, asynchronous, and anonymous youth advisory board (YAB). The YAB’s evolution fostered participation during the adaptation of an HIV care mobile health application.

**Methods:**

YAB members, comprised of YLWH in South Texas, engaged in the mobile application’s formative evaluation, adaptation, and pilot implementation. We collected feedback via surveys and interviews, analyzed and integrated responses, tracked participation and YAB adaptations, and performed content analysis.

**Results:**

Driven by feedback, the YAB evolved from in-person group meetings to the current iteration. We administered five surveys, and YAB members provided feedback on communication preferences; mobile app elements; privacy and confidentiality; and virtual support groups.

**Discussion:**

Our adaptive process highlights three primary drivers of innovation: COVID-19 risk reduction, asynchrony, anonymity. Our success in maintaining YAB engagement suggests the adapted model could be employed to support youth input in other contexts.

Youth and emerging adults living with HIV (YLWH) face challenges with care engagement. Targeted programs to support engagement in this age group are essential, and interventions are most successful when those affected participate in intervention design and implementation [1]. The Positive Youth Development (PYD) framework suggests that successful youth-focused interventions must incorporate youth voices [2]. However, few tested models exist for youth-focused research advisory boards [3].

Positive Links for Youth (PL4Y) uses the PYD framework to adapt an existing mobile health intervention, *PositiveLinks*, for YLWH 18- to 29-years-old in South Texas. *PositiveLinks* is a multicomponent mobile health intervention to improve retention in care for people living with HIV [4]. PL4Y will serve a younger population in a specific sociocultural context, so we convened a youth advisory board (YAB) to engage YLWH in intervention design and implementation. The YAB structure evolved to meet expressed needs of YLWH, to support participation, and to foster YLWH voice. This brief focuses on the emergence of a novel YAB structure, which may help others wishing to engage youth or communities preferring an anonymous, virtual structure (The impact of YAB members’ contributions to the mHealth intervention adaptation is the topic of a separate paper which details the adaptation process for the intervention).

## Methods

The original YAB design included quarterly in-person group meetings with compensation for meeting attendance. The YAB’s charge was to provide feedback on study design and intervention adaptation. The research team recruited members through community-based organizations, fliers distributed in HIV clinics, and collaborations with YLWH advocates. Inclusion criteria required members to be 18–29 years old and living with HIV in San Antonio, Texas. No research training was required, and each member was oriented to the study and its goals by the research team.

A team member collected feedback from YAB participants through email, phone call, text, or surveys and documented individual communication preferences for the following: in-person or virtual meetings, flexible meeting times, and considerations to protect anonymity of YAB members. We tracked the level of participation through attendance records and survey responses. The UT Health Science Center Institutional Review Board (IRB) judged these activities to be program evaluation and not human subject research.

## Results

The original YAB structure involved in-person, group meetings at a community-based site. In March 2020, the IRB suspended in-person interactions with research participants to reduce COVID-19 risk. In addition, prospective YAB members expressed a strong preference for virtual meetings due to scheduling constraints, transportation barriers, and anonymity concerns. This prompted a series of adaptations in response to YAB members’ expressed needs (Table). The original YAB iteration was composed of five members and majority identified as male (80%), Latinx (100%), and LGBTQIA+ (80%), with a median age of 22.8 years.

In our first adaptation, YAB meetings transitioned to a virtual platform but continued as scheduled group meetings. YAB members wanted to participate in the virtual YAB meetings without video and not have their names shown in the call. Attendance remained a challenge and reported reasons for low attendance included work and school obligations, busy schedules, abnormal sleep schedules, and forgetting about the meetings.

In the second adaptation, our team implemented an asynchronous model due to challenges in scheduling group meetings. This allowed members to provide individual feedback at convenient times and locations. We created Surveys One and Two, composed of free-response questions, in a word document, and emailed to members. However, YAB members noted challenges responding to questions within a Word document and difficulty completing surveys on cell phones.

In our third adaptation, we moved survey questions to Qualtrics, a responsive design platform, allowing youth to access the surveys via email or text and respond to surveys on cell phones. We redesigned shorter surveys with direct language to promote understanding. We re-created Survey Two using Qualtrics and had improved response rates.

Finally, a fourth adaptation asked YAB members to self-assign nicknames to identify individual perspectives longitudinally while maintaining anonymity. Currently, we seek quarterly feedback from YAB members through Qualtrics, allowing for asynchronous responses from members across digital platforms. YAB members respond to surveys using nicknames not linked to their identity, thus protecting member confidentiality and allowing for longitudinal assessment of member responses. Summaries of survey results, with key responses identified by nickname when appropriate, are provided to YAB members after each survey so that YAB members can respond to comments and see others’ reaction to their input. The fourth and current adaptation has 14 members and majority identify as male (71%), Latinx (71%), and LGBTQIA+ (79%), with a median age of 24.6 years. Most YAB members were in HIV care in San Antonio, and one member was a staff member at a community HIV clinic.

## Discussion

We iteratively adapted a traditional advisory board structure over 18 months in the context of COVID-19 and in response to YAB members’ requests for scheduling flexibility, anonymity, and mobile-friendly communications. This process led to the development of a novel structure: an asynchronous, virtual, and an anonymous YAB.

Our adaptive process highlights three primary drivers of innovation: reduction of COVID-19 risk, asynchrony, and anonymity. Though the COVID-19 pandemic forced change to virtual meetings, this transition was preferred by YAB members after in-person meeting restrictions were lifted. Virtual meetings may be more accepted by this age group, who are digital natives and comfortable interacting in a virtual environment [1,5]. For programs seeking input of youth and emerging adults, virtual modes of interaction may be more acceptable. Virtual interactions may also facilitate engagement of participants with transportation barriers or those in rural communities.

Second, asynchrony became a driver of innovation when scheduling group virtual meetings was unsuccessful. YAB members described multiple challenges to synchronous scheduling, including lack of control over their schedules. YAB member input also led to adaptations to support survey responses on smartphones. We preserved feedback quality with an online survey tool that allows responses across multiple platforms. Asynchronous participation with an improved feedback approach was received positively and led to increased engagement, which may be relevant in other contexts when scheduling challenges arise.

Finally, many YAB members expressed concerns regarding disclosure of HIV status or sexual orientation. Studies demonstrate the profound negative impact of HIV-related stigma on care engagement [6–9]. The transition to an asynchronous YAB structure supported members’ anonymity. However, both asynchrony and anonymity pose challenges to dialogue between YAB members and the research team. The creation of nonidentifying nicknames for each YAB member facilitates tracking of responses over time and promotes YAB member ownership of ideas. For advisory boards within marginalized communities or focused on stigmatizing topics, protecting member identity is essential when establishing methods of communication. Our success in maintaining youth engagement with an asynchronous, virtual, anonymous advisory board suggests this model could support youth input in other contexts.

## Figures and Tables

**Table:** Iterative process of youth advisory board (YAB) adaptation over 18 months (March 2020 through September 2021), including strengths and challenges identified by YAB members and research team

Iteration^a^	Structure	Membership^b^	Participation	Strengths	Challenges
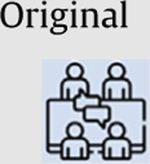	In-person group meetings	Five members	Cancelled for COVID-19 precautions	Face-to-face dialogue Brainstorming Existing models in literature	COVID-19 risk Transportation Confidentiality
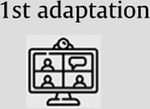	Virtual, synchronous, group meeting	Seven members	Meeting 1: 20.0% (1/5) Meeting 2: 33.3% (2/6) Meeting 3: 00.0% (0/7)	Face-to-face dialogue Brainstorming Flexible location	Fixed meeting times Confidentiality Low attendance
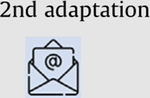	Virtual, asynchronous, responses to an emailed Word document	Nine members	Survey 1: 83.3% (5/6) Survey 2: 22.2% (2/9)	Flexible scheduling Flexible location	No engagement between members Low overall engagement (survey 2)
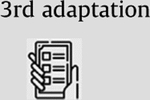	Virtual, asynchronous, responses on smartphone responsive survey platform	Nine members	Revised survey 2: 66.7% (6/9)	Flexible scheduling Flexible location Customizable engagement mode Survey access on smartphone	No engagement between members Required survey reminders
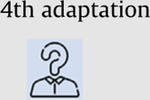	Virtual, asynchronous, anonymous responses on smartphone responsive survey platform	14 members	Survey 3: 50.0% (7/14) Survey 4: 15.4% (2/13) Survey 5: 69.2% (9/13)	Anonymous but allows for engagement between members Retains strengths of 1–3rd adaptation	Misunderstood nicknames Required survey reminders No face-to-face dialogue or brainstorming

a Icons made by Freepik from www.flaticon.com.

b Membership demographics only available at two timepoints. Gift cards were used as incentives for participation throughout all iterations.
